# Species-Agnostic Tools for HPAI H5N1 Antibody and Antigen Detection in Dairy Cows, Chickens, and Humans

**DOI:** 10.21203/rs.3.rs-10656302/v1

**Published:** 2026-09-13

**Authors:** Laura R.H. Ahlers, Nicholas Sammons, Brian S. Lane, Yeming Wang, Leonid Dzantiev, Anu Mathew, Jason Abraham, Ismaila Shittu, Lyudmyla V. Marushchak, Gregory C. Gray, Walter N. Harrington, Lisa Kercher, Ahmed Kandeil, Jacob N. Wohlstadter, George B. Sigal

**Affiliations:** Meso Scale Diagnostics, LLC; Meso Scale Diagnostics, LLC; Meso Scale Diagnostics, LLC; Meso Scale Diagnostics, LLC; Meso Scale Diagnostics, LLC; Meso Scale Diagnostics, LLC; Meso Scale Diagnostics, LLC; University of Texas Medical Branch; University of Texas Medical Branch; University of Texas Medical Branch; St. Jude Children’s Research Hospital; St. Jude Children’s Research Hospital; St. Jude Children’s Research Hospital; Meso Scale Diagnostics, LLC; Meso Scale Diagnostics, LLC

**Keywords:** influenza H5N1, bridging serology, nucleoprotein antigen, multiplex, HPAI, human health

## Abstract

High Pathogenicity Avian Influenza (HPAI) A (H5N1) circulates in wild birds, causing outbreaks in domestic poultry, but recently became enzootic in the United States spilling over into various mammals, including cattle. The zoonotic potential of H5N1 clade 2.3.4.4b emphasizes the need for tools for surveillance in mammalian and avian species. We developed two species-agnostic assays using the MESO SCALE DISCOVERY^®^ (MSD) platform to enhance current surveillance methods: a sandwich immunoassay for influenza A nucleoprotein (NP) antigen, and a bridging serology assay for influenza A/H5 specific antibodies. We assessed the assays by testing milk and serum from cattle herds experiencing H5N1 incursions, serum from H5N1-vaccinated humans and chickens, and negative control samples from cattle, chickens, and humans.

Antibodies and antigen were detected in milk and serum from H5-exposed cattle herds, but not in samples from putatively naïve herds. The sensitivity for identifying exposed animals improved by combining antigen and antibody testing. The bridging serology assay accurately differentiated serum from H5-vaccinated chickens and humans, relative to H5-naïve controls. High specificity in human samples was achieved by using H1 hemagglutinin to block potentially cross-reactive activity. The studies demonstrate the applicability of these methods to diverse species and facilitate their use in surveillance studies.

## INTRODUCTION

High Pathogenicity Avian Influenza (HPAI) influenza A (H5N1) circulates globally and causes significant mortality in bird populations,^1^ but in recent years has been increasingly associated with morbidity and mortality in various mammalian species due to spillover events. The current pan-zoonotic is primarily driven by HPAI H5N1 clade 2.3.4.4b viruses, a clade that has expanded since 2020. ^2^ The clade was first identified in North America in 2021 and rapidly assorted with circulating low pathogenicity avian influenza (LPAI) viruses and subsequently dominates every continent on which HPAI has been detected.^3,4^ H5N1 infections have been identified in unconventional and varied mammalian hosts including dairy cows,^5^ farm cats,^6,7^ marine wildlife,^8–10^ and terrestrial wildlife.^11^ Infections in farm animals, such as dairy cows, are of special concern because of their economic significance and because of the increased risk of zoonotic transmission to agricultural workers.^12^ The potential risks emphasize the need for surveillance to identify H5N1 virus spread in human and animal populations. Due to the complex ecosystem of H5N1 virus transmission that spans avian and mammalian species, there is a need for simple and scalable assays for identifying H5N1 infections that are species-agnostic and can be applied to samples from a wide range of animals without reformulation or continuous optimization.^13,14^

Influenza infections may be identified in biological samples by detecting the presence of viral nucleic acids or proteins.^15^ Due to the availability of nucleic acid amplification technologies such as PCR, nucleic acid detection is typically more sensitive; however, antigen detection can provide advantages through faster protocols, lower cost and amenability to use in simple point-of-care devices such as lateral flow devices. Nucleic acid and antigen detection are extremely useful as screening tools to identify active H5N1 infections or to confirm H5N1 infections in a cluster of symptomatic cases. However, they are only useful during active infection, limiting their utility in general population surveillance. Complementarily, the prevalence of infection across a population can be surveyed through the use of serology assays to detect host antibodies generated in response to infection.^16–18^ While serological assays are less sensitive early in infection, they provide a broader picture by identifying current and past infections within a population. Serological surveillance is typically carried out using indirect ELISA assays, which can be automated and multiplexed, but requires species-specific detection reagents and may suffer from significant cross-reactivity between strains within influenza types or subtypes. Cross-reactivity is of special concern for influenza H5 surveillance in human populations due to the high prevalence of infections and vaccinations with the related H1 subtype.^19–21^ In influenza studies, serological studies often focus on antibodies capable of blocking viral cell entry and infection, employing techniques such as microneutralization (MN) and hemagglutination inhibition (HAI) assays. These assays are more strain-specific than indirect serology, but are less practical to perform, requiring live virus and biological materials such as live cells that pose challenges for safety, storage, and consistency.

To provide tools for rapid high-throughput surveillance for influenza H5N1 infections, we developed species-agnostic assays, employing Meso Scale Discovery’s (MSD’s) sensitive electrochemiluminescence (ECL) assay platform, for detecting influenza A nucleoprotein (NP) antigen and influenza A/H5 specific antibodies in a variety of sample matrices. The NP assay utilizes a sandwich immunoassay format employing two monoclonal antibodies for antigen capture and detection (Fig. 1A). The assay to measure H5 antibodies utilizes a bridging serology format (Fig. 1B) and, for samples expected to have high anti-H1 activity, an H1 blocker to provide high specificity in a simple scalable format. We assessed the utility of the assays by testing milk and serum from H5N1 affected dairy cattle herds, serum from H5N1 vaccinated humans and chickens, and negative control samples from the three species.

## RESULTS

### H5 Antigen and Antibodies in Milk from H5N1-Exposed Dairy Cows

We tested dairy cow milk from H5N1- affected herds for influenza A RNA (Fig. 2A), nucleoprotein (NP) antigen (Fig. 2B) and H5-specific antibodies (Fig. 2C), and compared the measured NP antigen and H5 antibody levels to milk from putatively H5N1 naïve control cows. Of the 50 H5N1-affected herds cow milk samples, 31 (62%) were positive by qRT-PCR and 26 (52%) were positive for NP antigen. Antigen detection generally correlates with nucleic acid detection: 18 of 19 qRT-PCR negative samples were negative for NP antigen, while 23 of 31 qRT-PCR positive samples were positive for NP antigen. In the qRT-PCR positive samples with positive antigen results, the antigen concentrations could be extremely high with measured concentrations ranging from 1.3 ng/mL to 20 μg/mL, relative to our cut-off concentration of 0.57 ng/mL (paired Ct values for the same samples range from 15.7 to 35.9 cycles). One qRT-PCR negative sample had a positive antigen result of 85.5 ng/mL NP, indicating that this result is likely to be a true positive for the presence of antigen (Ct value = 40.1 cycles). None of the 30 milk samples from H5N1 naïve cows were positive for antigen.

We then determined the amount of H5-specific antibodies present in each cow milk sample (Fig. 2C). A total of 31 of 50 (62%) milk samples from H5N1-affected cows were positive for antibodies to H5. The percentage was much higher for qRT-PCR negative samples – 18 of 19 (95%) – than for qRT-PCR positive samples – 13 of 31 (42%). None of the 30 milk samples from H5N1 naïve cows were positive for antibody.

Figure 2D shows the correlation of measured antigen and antibody concentrations and clusters the samples into groups based on whether antigen and/or antibody was detected: 36% of samples had detectable antigen, but no measured antibodies; 46% of samples had detectable antibodies, but no measured antigen; and 16% had some amount of both antibodies and antigen. Only one milk sample from the H5N1-affected cows had neither detectable antibody nor detectable antigen.

### H5 Antigen and Antibodies in Serum from H5N1-Exposed Dairy Cows

We tested serum from H5N1-affected cows and putatively naïve control cows for H5 antibodies using the conventional microneutralization (MN) assay (Fig. 3A) and the H5 Bridging Serology assay (Fig. 3C). The same samples were also tested for NP antigen (Fig. 3B). Only one serum sample (an MN-negative sample from an H5N1 affected cow) was positive for NP antigen. The H5 Bridging Serology assay detected H5-specific antibodies in all 11 MN-positive serum samples, as well as in an additional 12 serum samples from the 49 H5N1-affected cows that were not positive by MN. The measured antibody levels by bridging serology in the 11 MN-positive samples [median (IQR) = 515 (350, 1717) AU/mL] was significantly higher than in the 12 MN-negative samples [median (IQR) = 3.851 (1.247,7.997) AU/mL] (Mann-Whitney test, *p* < 0.0001). For the 11 MN-positive samples, we observed a strong correlation (Pearson R = 0.711) of MN titer with H5 antibody concentration from bridging serology (Fig. 3D). None of the 38 serum samples from putatively H5N1-naïve cows were positive for antigen or antibody.

### H5 Antibodies in Serum from Vaccinated Chickens

We tested serum from chickens vaccinated against H5 for H5-specific antibodies using the H5 Bridging Serology assay. The samples came from 30 chickens in six groups of 5 chickens: five groups received different vaccine formulations (Fig. 4A) and the remaining group was a placebo control. All 25 samples from vaccinated chickens were positive for H5-specific antibodies, with each vaccine type inducing differing levels of antibody responses (Fig. 4B). None of the 5 control chicken samples were positive for H5-antibodies.

### H5 Antibodies in Serum from Vaccinated Humans

We also measured antibody responses to H5N1 in four pooled human serum samples from participants in experimental H5N1 vaccine studies and 91 human serum samples from healthy controls (Fig. 5A). The vaccine incorporated antigens from the older H5N1 clades 1 (A/Vietnam/1203/2004) and 2.1.3.2 (A/Indonesia/05/2005), but we anticipated that the serological responses will be cross-reactive with the clade 2.3.4.4b antigen used in our serology assay.^22^ The control sera are presumed naïve due to the low prevalence of H5N1 in the human population.

Figure 5B shows H5 antibody levels measured with the H5 bridging serology assay with and without the inclusions of the H1 Cross-Reactivity Blocker, a soluble H1 construct that can be added to the assay diluent to block H1-specific antibodies that could potentially cross-react with H5. In the absence of the blocker, we measured strong antibody activity from the vaccinated samples (concentrations ranging from 49 to 206 AU/mL), including a sample with a relatively weak HAI titer (HAI = 20, data from BEI Resources) for its cognate vaccine target, and presumably weaker titer for the clade 2.3.4.4b antigen used in the bridging assay. A number of putatively naïve samples had detectable antibody concentrations, presumably from cross-reacting antibodies resulting from non-H5 influenza infections. Most of these had concentrations well below those of the positive samples, but three putatively naïve samples had concentrations that were greater than 5 AU/mL and in the same range as the positive samples. Addition of the H1 blocker had only a weak effect on the measured antibody levels for the positive samples. However, the blocker greatly reduced the measured concentrations for the three outlier negative samples, bringing all the measured concentrations for the putatively negative samples below 5 AU/mL, and providing a complete separation of the positive and negative samples.

## DISCUSSION

Because of the diverse species that are infected with influenza H5N1, there is a need for assays that are species-agnostic and can address hypotheses regarding infection status and immunity to the virus. For this reason, we developed assays to measure NP antigen and H5 antibodies and tested them with samples from cattle, chickens, and humans. Milk is a non-invasive sample type, is rich in antibodies, and is convenient to collect. HPAI H5N1 replicates effectively in the mammary gland of dairy cattle, and this is an important transmission route to mitigate, as well as a critical sample matrix for monitoring influenza infections.^23,24^ We detected anti-H5 antibodies and influenza NP antigen in the milk from H5N1-affected dairy cows, but not in samples from putatively naïve cows, highlighting the potential utility of applying our assays to milk samples for H5N1 surveillance studies. We found that the NP antigen could be measured in milk samples from H5N1-exposed cows at levels as high as 19 μg/mL. The presence of NP antigen generally correlated with the presence of influenza RNA, and is consistent with observations of infective virus in milk^5,25^ potentially resulting from virus-infected cells in the cow udders. Our detection of anti-H5 antibodies in milk of H5N1-exposed cows is also consistent with prior studies: After the spread of H5N1 influenza in US dairy herds, H5-reactive antibodies were detected in store-bought cow milk,^26^ and these antibodies were shown to have antiviral activity. Antibodies have also been detected in cow milk following H5N1 vaccination,^27^ indicating that that serology in this sample type may be useful for detecting both prior infections and vaccination. Our results suggest that combining antigen and antibody detection for analyzing milk samples may be particularly advantageous. Our testing of milk samples from H5N1-exposed cows identified (i) a number of samples with detectable antigen but not antibody, presumably because the cows had acute infections and had not yet developed a strong antibody response and (ii) a number of samples with detectable antibody but not antigen, presumably because the cows had developed an antibody response and cleared the infection. All but one of the milk samples from H5N1-exposed cows had either detectable antigen or detectable antibody, showing that combining the two assays has the potential to increase the sensitivity for detecting infection across the time course of infection and recovery.

Serological testing of cow serum samples has been used extensively in H5N1 surveillance studies in dairy and cattle herds,^28^ but these studies have typically used serology techniques such as HAI or MN that require the use of live virus. In testing of serum samples from H5N1-exposed dairy cows, we demonstrated that our H5 bridging assay correlated strongly with MN. Furthermore, the H5 bridging assay detected H5 antibodies in a number of MN-negative samples from H5N1-exposed cows. The measured concentrations in these samples were lower than in the MN-positive samples. This result, combined with the high observed specificity in putatively naïve samples, suggests that the bridging serology assay can provide higher sensitivity for detecting exposures than MN.

Birds are routinely surveyed for the presence of avian influenzas: Wild birds are monitored for the spread of HPAI H5N1,^29^ and US poultry producers test to provide early detection of infection in domestic flocks^30^, identify infectious agents, and use this information to inform policy proactively. We demonstrated that the same H5 bridging serology assay used to detect anti-H5 antibodies in mammalian samples could accurately differentiate chicken serum samples from naïve and H5-vaccinated chickens. The assay was able to detect H5 antibodies in chickens vaccinated with EMULMAX and I.A. Plus, vaccines that generally produce undetectable titers by HAI and MN.^31^ Interestingly, Brice, et al. determine that despite the lack of HAI and MN titers following vaccination, these two vaccines are protective against H5N1 challenge and have similar survival rates to Vaxigen, AVILAB, and the experimental H5N1 vaccine. This result provides additional evidence that the bridging serology assay is more sensitive than HAI and MN methods, and antibodies detected with the assay are biologically relevant. Our work here demonstrates feasibility for measuring H5N1-exposure in bird populations, such as chicken flocks, with limited influenza exposures. Further work is needed to demonstrate accurate performance in wild bird populations that are likely to have more varied prior exposures to different influenza types and strains, and to evaluate more conveniently collected samples such as oropharyngeal and cloacal swabs.

Measurement of anti-H5 antibodies in serum samples, typically by HAI or MN, has been used in human surveillance studies looking to identify the potential for zoonotic spread of H5N1 from farm animals to farm workers or other people who come in close contact with these animals. Serosurveillance of persons with occupational exposure to potentially infected cows identified 8 of 115 dairy farm workers (June-August 2024) and 3 of 150 bovine veterinarians (September 2024) as having antibodies to H5, as measured by MN and HAI.^32,33^ One problem associated with many H5 surveillance studies is the lack of comparison to a low-exposure risk population to build confidence that positive determinations are associated with exposure. Given the high prevalence of the related Type A Group 1 H1 HA subtype in human populations,^19–21^ it is important in H5 surveillance studies to rule out false positives due to cross-reacting anti-H1 antibody activity. In one study of two dairy workers that tested positive for H5N1 by qRT-PCR,^34^ MN and HAI detected positive anti-H5 antibody responses that remained high if the samples were pre-adsorbed with H1 and H3 HAs to block potential cross-reacting antibodies from endemic human influenza strains. Daniel, et al. similarly identified that H5N1-naïve individuals develop antibodies to H5 that a cross-reactive to H1, primarily on epitopes on the HA stem.^35^ In our work, we used a similar blocking approach using an H1 HA blocker, but applied it to the simpler and more scalable bridging serology format. We demonstrated the ability to detect antibodies in serum samples from human vaccine studies, as a surrogate for infection. We also characterized the assay with a set of 91 normal, putatively H5-naïve human serum samples and showed that, in three samples that provided high outlier concentrations in the absence of the H1 blocker, the measured concentrations in the presence of the H1 blocker were reduced to levels well below the positive vaccine samples. The results suggest that, with the H1 blocker and an appropriate cut-off value, the bridging serology assay can achieve sufficient specificity to be used in surveillance studies for human H5 exposure.

In this work, we demonstrate the use of an ECL-based species-agnostic sandwich immunoassay and bridging serology assay for measuring Influenza A NP antigen and H5-specific antibodies in a variety of sample types from different species. Strengths of the work include the inclusion of samples from three different species and comparison of samples from putatively positive and negative populations. The main limitations are associated with the sample sets from H5 exposed animals and people that were readily available for use in assay characterization. We used milk and serum samples collected from dairy cows in H5N1-affected herds, but we had limited data on the infection status of the individual animals. We did not have access to samples from H5N1-infected humans or chickens, and used samples collected after H5N1 vaccination as surrogates. The bridging serology assay has been shown by others to measure H5-specific antibodies in confirmed cases in humans and birds.^36^ Further work including samples from individuals with well-characterized infections is needed to definitively establish assay cutoffs and determine assay sensitivity and specificity in the different species.

As the current HPAI H5N1 outbreak continues to progress, we look towards a One Health approach, in which human and animal health are considered together. Our work demonstrates species-agnostic tools to aid in research of the diverse animal hosts of H5N1. Collectively, these tools can assist in serosurveillance and virus detection to support wildlife, animal welfare, the food supply, and human health.

## METHODS

### Ethics Statement

We confirm that all experiments in this study were carried out in accordance with relevant guidelines and regulations. Protocols for collection of blood and milk from cattle were reviewed by the Chair of the Institutional Animal Care and Use Committee (IACUC) at the University of Texas Medical Branch and viewed as exempt from formal IACUC ethical review. Chicken serum samples came from a previously conducted chicken vaccination study that was approved by the St. Jude Children’s Research Hospital IACUC (protocol number 428). All human samples were de-identified, archived samples sourced from publicly available bio-repositories that had been collected with appropriate donor consent under protocols reviewed by an Institutional Review Board (IRB); normal human serum samples were purchased from BioIVT; pooled human serum from H5 vaccinated individuals were provided by BEI Resources.

### Cattle Samples

Milk (n = 50) and serum (n = 60) samples from cattle herds with current and recent H5N1 epizootics were collected in 2024 and 2025 from herds in New Mexico, South Dakota and Texas by the University of Texas Medical Branch (UTMB). The milk and serum samples were previously tested at the UTMB, where their H5N1 antigen and antibody status was first identified. The serum and milk samples were unmatched, i.e., not collected from the same cows. The samples were gamma irradiated at the UTMB to remove any potential live virus prior to shipment to MSD for additional antigen and serologic testing.

The healthy cow specimens came from Purdue University and Oklahoma State University. Milk (n = 30) and unmatched serum (n = 30) samples from Purdue were collected in Indiana in 2025 from healthy animals in herds not known to suffer H5N1 incursions. Serum samples (n = 8) from Oklahoma were collected pre-2024, and prior to the spread of H5N1 in US cattle as part of an unrelated study. These samples were collected from four crossbred female dairy calves prior to and 28 days post infection with the Cooper strain of bovine herpes virus 1 (BoHV-1) in 2020.^37^ BoHV-1 is a DNA virus unrelated to the RNA virus influenza A.

### Chicken Samples

Specific pathogen-free (SPF) chickens were hatched at the ARC Holding Animal Lab at St. Jude Children’s Research Hospital. Chickens were vaccinated intramuscularly at 3 weeks of age with the following vaccines: I.A. PLUS (A/chicken/Mexico/232/94 (H5N2)), EMULMAX AI + N5 G15 (A/pollo/Guanajuato/20966/15 (H5N2)); Vaxigen Flu H5N8 (rg-A/green winged teal/Egypt/877/2016 (H5N8)), AVILAB REVGEN 51 (A/Falco_rusticollus/EdoMex/CPA-19638-22/2022 (H5N1)), and an experimental vaccine made from rg-A/Bald eagle/FL/W22-134-OP/2022 (H5N1) (HA modified, NA) + PR8.^31^ Serum was collected 4 weeks following vaccination for testing for influenza antibodies.

### Human Samples

The following reagents were obtained through BEI Resources, NIAID, NIH: (1) Human Reference Antiserum to Influenza A/Indonesia/05/2005 (H5N1), Low Titer, NR-33667; (2) Human Reference Antiserum to Influenza A/Indonesia/05/2005 (H5N1), Medium Titer, NR-33668; (3) Polyclonal Anti-Monovalent Influenza Subvirion Vaccine rgA/Vietnam/1203/2004 (H5N1), (antiserum, Human), Low Titer Pool, NR-4110; and (4) Polyclonal Anti-Monovalent Influenza Subvirion Vaccine rgA/Vietnam/1203/2004 (H5N1), (antiserum, Human), High Titer Pool, NR-4109. The Certificates of Analysis from BEI includes HAI assay titers for the samples measured using the vaccine target strain. Control human sera were obtained from BioIVT from 91 healthy donors. The samples were collected with appropriate donor consent under protocols reviewed by an Institutional Review Board (IRB). These samples are presumed naïve due to the low rates of H5N1 infection in the general human population.^38^

### qRT-PCR Assay

At UTMB, the cattle milk specimens were tested for molecular evidence of influenza A matrix gene RNA by qRT-PCR assay as previously reported.^39,40^ Samples with cycle threshold (Ct) of less than 38 cycles were considered positive .

### Microneutralization Assay

Cattle serum samples were tested for H5 neutralizing antibody with a microneutralization (MN) assay using a recombinant H5N1 virus (rg-A/bald eagle/Florida/W22-134-OP/2022 of clade 2.3.4.4.b) as previously described. ^41^ Briefly, samples underwent two-fold serial dilutions and were incubated with 100 TCID_50_ of the recombinant virus for 1 hour. The mixture was then transferred onto a near confluent monolayer of Madin-Darby canine kidney cells in a 96-well plate. Following an 18–20 hour incubation, anti-NP protein expression was detected using mouse monoclonal IgG antibody (Sino Biological, Wayne, PA, USA) and horse-radish peroxidase conjugated goat anti-mouse IgG (Thermo Fisher Scientific, Waltham, MA, USA) as secondary antibody. Samples were considered to possess neutralizing antibodies, if the MN titer was ≥ 1:40.

### Influenza A NP Assay

Antigen detection was performed using the R-PLEX Human Influenza A NP Assay from MSD (K1519GR) per the protocol in the product insert. Assays are carried out in an MSD Gold 96-Well Small Spot Streptavidin SECTOR^™^ Plate, which provides streptavidin coated electrodes within each well to support ECL measurements. In brief, the protocol involves the following steps: (i) pre-washing the plate then adding 25 μL of biotin-labeled anti-NP capture antibody in MSD^®^ Diluent 100 and incubating to immobilize the capture antibody; (ii) washing the plate then adding 25 μL of MSD Diluent 11 and 25 μL of sample to the wells, and incubating to bind analyte from the sample; (iii) washing the plate and adding 50 μL of anti-NP detection antibody labeled with the MSD SULFO-TAG^™^ ECL label, and incubating to form a sandwich complex; and (iv) washing the plate, adding 150 μL of MSD GOLD Read Buffer B and measuring ECL on an MSD MESO**®** SECTOR S 600M or QuickPlex^®^ SQ 120MM ECL plate reader. All incubations are one hour with shaking, for a total assay time of about three hours. Serum samples were tested undiluted and milk samples were diluted 1:10 in MSD Diluent 11. Samples that were too high to quantitate at the default dilution for a sample type were retested at higher dilution, as needed, to produce signal in the linear dynamic range of the assay. Samples were quantitated relative to a calibration standard, a purified type influenza A virus preparation provided with the kit. The calibration standard was assigned in units of ng of influenza A/Wisconsin/67/2022 nucleoprotein per mL using a recombinant protein preparation (Sino Biological 40942-V08B).

### Hemagglutinin (HA) Antigens

The H5 and H1 antigens used in the H5 Bridging Serology Assay are HA head domains expressed in a mammalian expression system. The H5 HA used as an antigen in the assay is derived from the sequence for the HA of the clade 2.3.4.4b H5N1 strain A/chicken/Ghana/AVL-763_21VIR7050-39/2021. The H1 HA used as a blocker is derived from the sequence for the HA of the H1N1 strain A/Hawaii/70/2019. Both proteins were expressed with a C-terminal His6 purification tag.

### H5 Bridging Serology Assay

Antibodies specific to Influenza A H5 were measured using the Influenza H5 Bridging Serology Assay from MSD (K150AXJU) per the protocol in the package insert. The protocol involves an off-line incubation step to form the bridging complex, followed by capture and detection of the complex in MSD GOLD 96-Well Small Spot Streptavidin SECTOR Plates. In brief, the protocol involves the following steps: (i) combining 40 μL of sample with 40 μL of a Master Mix containing biotin-labeled H5 antigen and SULFO-TAG labeled H5 antigen in MSD Diluent 100 in a polypropylene 96-well plate and incubating 1 hour with shaking; (ii) transferring 50 μL of the resulting solution to a pre-washed MSD GOLD 96-Well Small Spot Streptavidin SECTOR Plates and incubating 1 hour with shaking; and (iv) washing the plate, adding 150 μL of MSD GOLD Read Buffer B and measuring ECL on an MSD MESO SECTOR^®^ S 600M or MESO QuickPlex SQ 120MM ECL plate reader. Total time for the assay is about two hours. Samples were diluted 1:10 (cow milk and chicken serum), 1:20 (human serum), or 1:100 (cow serum) in MSD Diluent 100 Samples were quantitated relative to the high antibody control as a calibration standard. A seven-point dilution series of the standard was generated by 1:4 serial dilution of the calibration standard in Diluent 100. Each dilution and a diluent blank were run in duplicate on each assay plate. The top calibration standard was assigned a concentration of 110 arbitrary units (AU) per mL.

In some experiments, the formation of the bridging complex was carried out in the presence of H1 antigen as a blocker to prevent signal from any anti-H1 antibodies in the sample with strong H5 cross-reactivity. These measurements were conducted using the same protocol described above, except that sample dilution was carried out in Diluent 100 containing the H1 blocker (R93BL). Results presented for the H5 Bridging Serology assay were based on testing carried out without H1 blocking, unless specifically indicated in the text.

### Sample Quantitation

For both Influenza NP and H5 Bridging Serology assays, samples were quantified to the kit calibrator using a 7-point calibration curve and four-parameter-logistic (4PL) fit with 1/Y^2^ weighting, following the product inserts. Samples that quantified at or below the limit of detection (LOD) were set to the LOD value, with LOD calculated as the concentration (based on the calibration fit) that gives a signal 2.5 standard deviations above the signal for a blank sample. The LOD for the Influenza NP assay was 0.029 ng/mL. The LOD for the H5 Bridging Serology assay was 0.001432 AU/mL. Reported concentrations were corrected for sample dilution. The cut-points for identifying samples with “positive” antigen or antibody activity were set to 2 times the LOD, after correcting for the default sample dilution for the sample type. The cut-points for the Influenza NP assay were 0.0574 ng/mL for cow serum and 0.574 ng/mL for cow milk. The cut-points for the H5 Bridging Serology Assay were 0.0286 AU/mL (cow milk and chicken serum), 0.0573 AU/mL (human serum) and 0.286 AU/mL (cow serum).

## Figures and Tables

**Figure 1:**
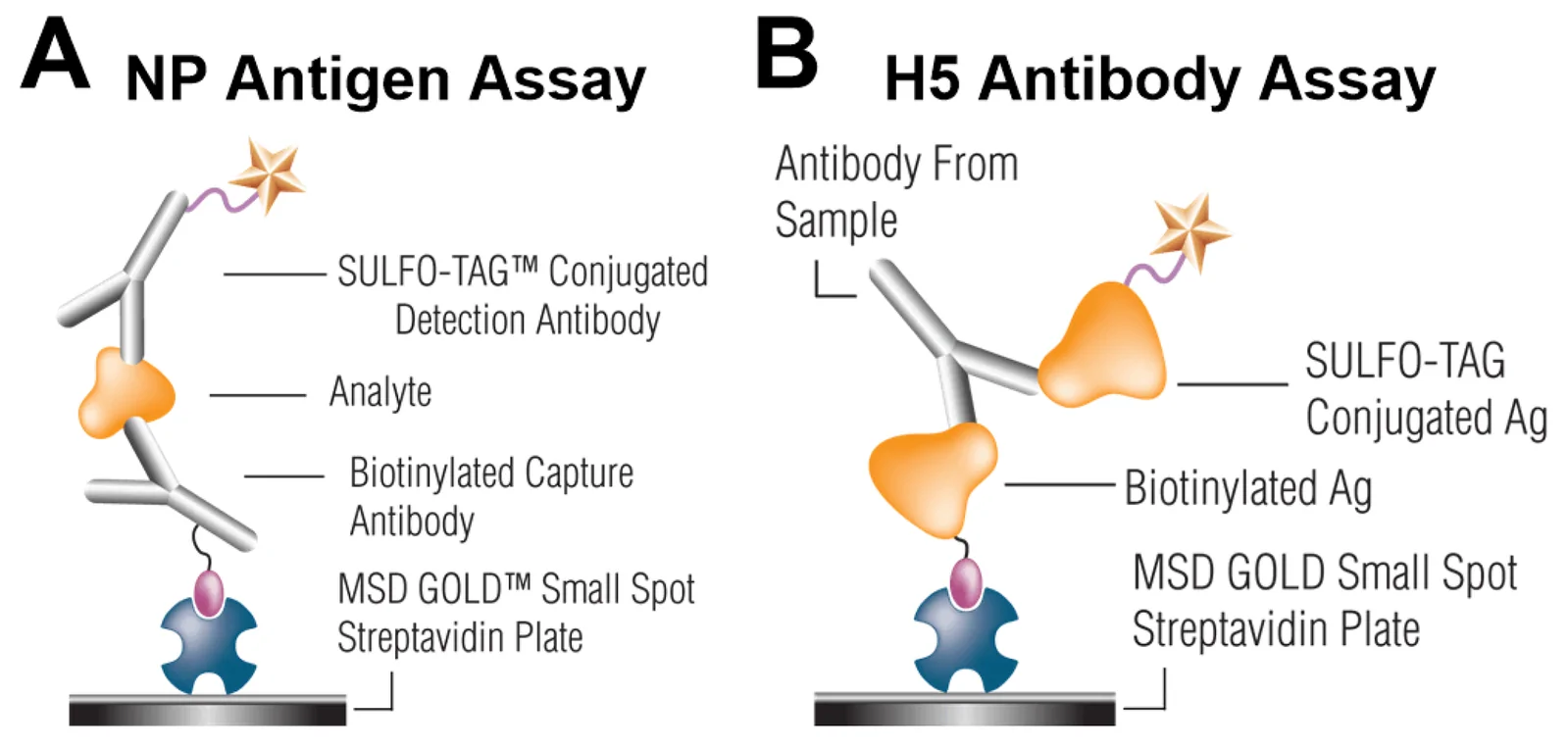
Species-agnostic assays for measuring antigen and antibodies from samples. (A) R-PLEX sandwich immunoassay for nucleoprotein antigen detection. (B) H5 Bridging Serology assay for the measurement of H5-specific antibodies.

**Figure 2:**
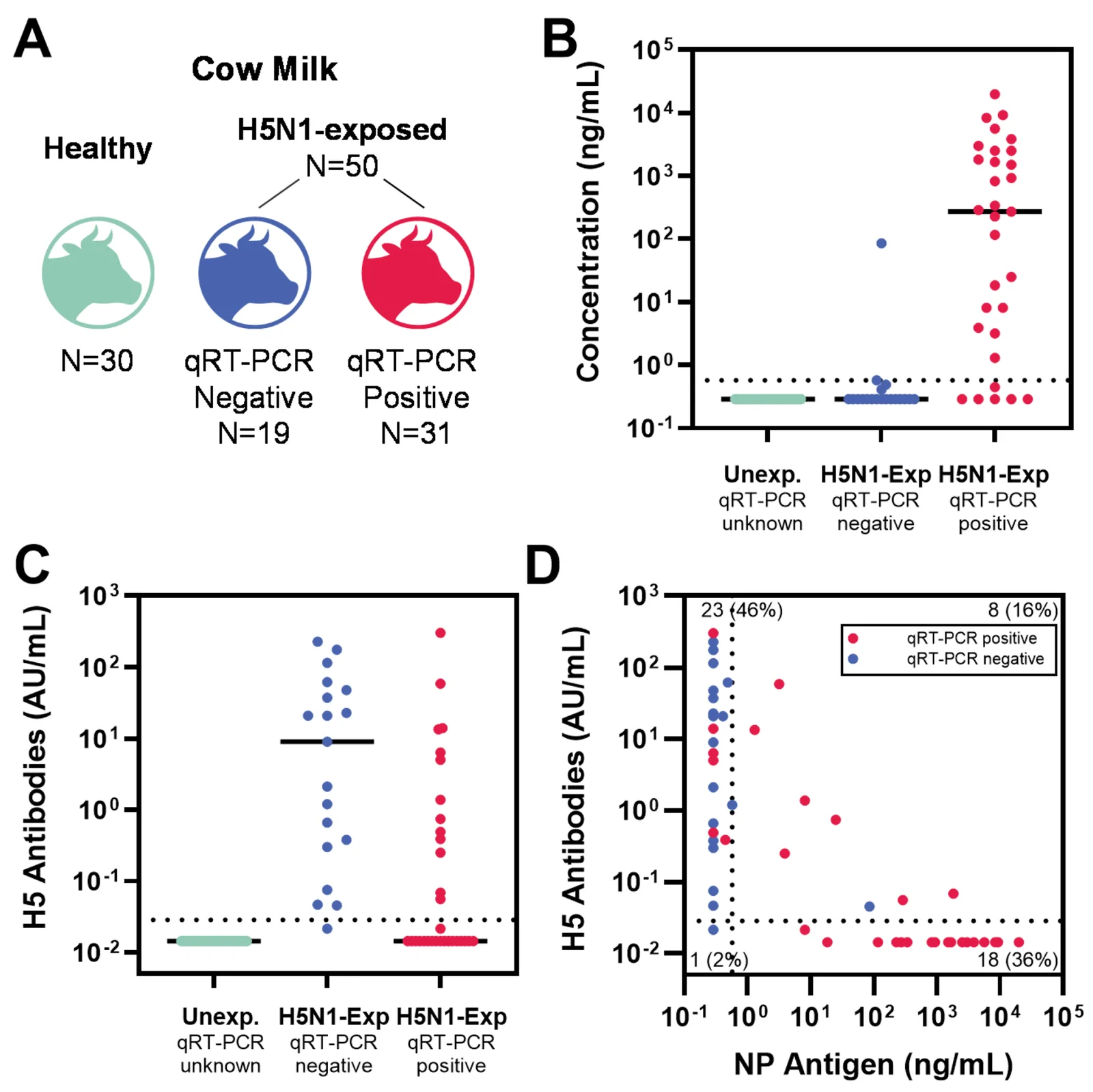
Dairy cow milk contains NP antigen and H5-specific antibodies. (A) The sample set of the study includes 30 healthy control milk samples and 50 H5N1-exposed cow milk samples that were tested for influenza nucleic acid by qRT-PCR. (B) Concentration of influenza NP in cow milk. (C) Concentration of H5-specific antibodies. (D) Correlation of antibody measurements vs. NP antigen concentration. Dots represent individual values and solid horizontal lines represent the median for that group. The horizontal and vertical dashed lines are the cut-off values (2 x LOD) used to identify positive samples.

**Figure 3:**
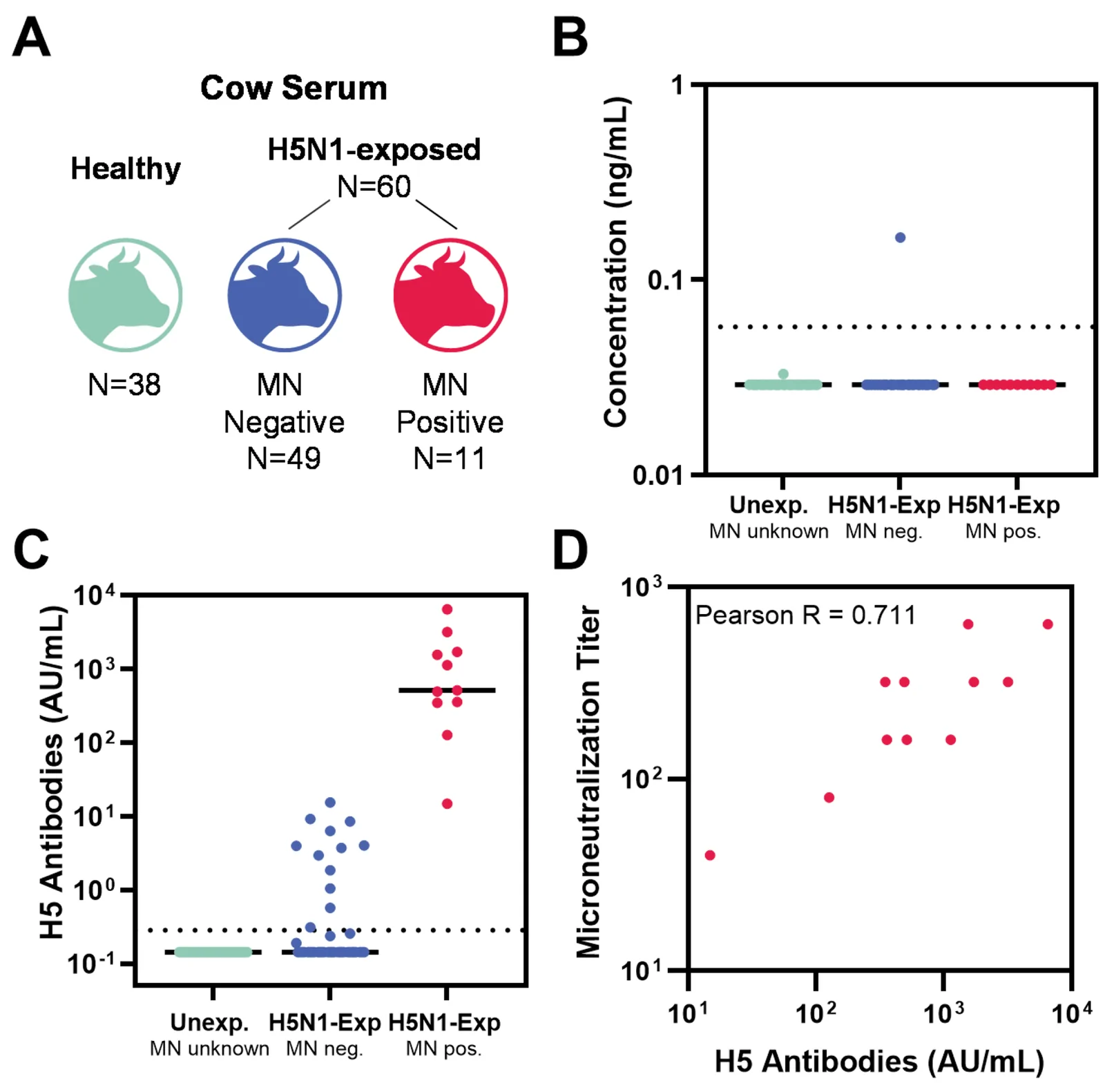
Dairy cow serum contains H5-specific antibodies that correlate with microneutralization titer. (A) The sample cohort of the study includes 38 healthy control serum samples and 60 H5N1-exposed cow serum samples, which were tested by microneutralization (MN) assay. (B) Concentration of NP in cow serum. (C) Concentration of H5-specific antibodies. (D) Correlation of microneutralization titer vs. antibody concentration. Dots represent individual values and solid horizontal lines represent the median for that group. The horizontal dashed lines are the cut-off values (2 x LOD) used to identify positive samples.

**Figure 4:**
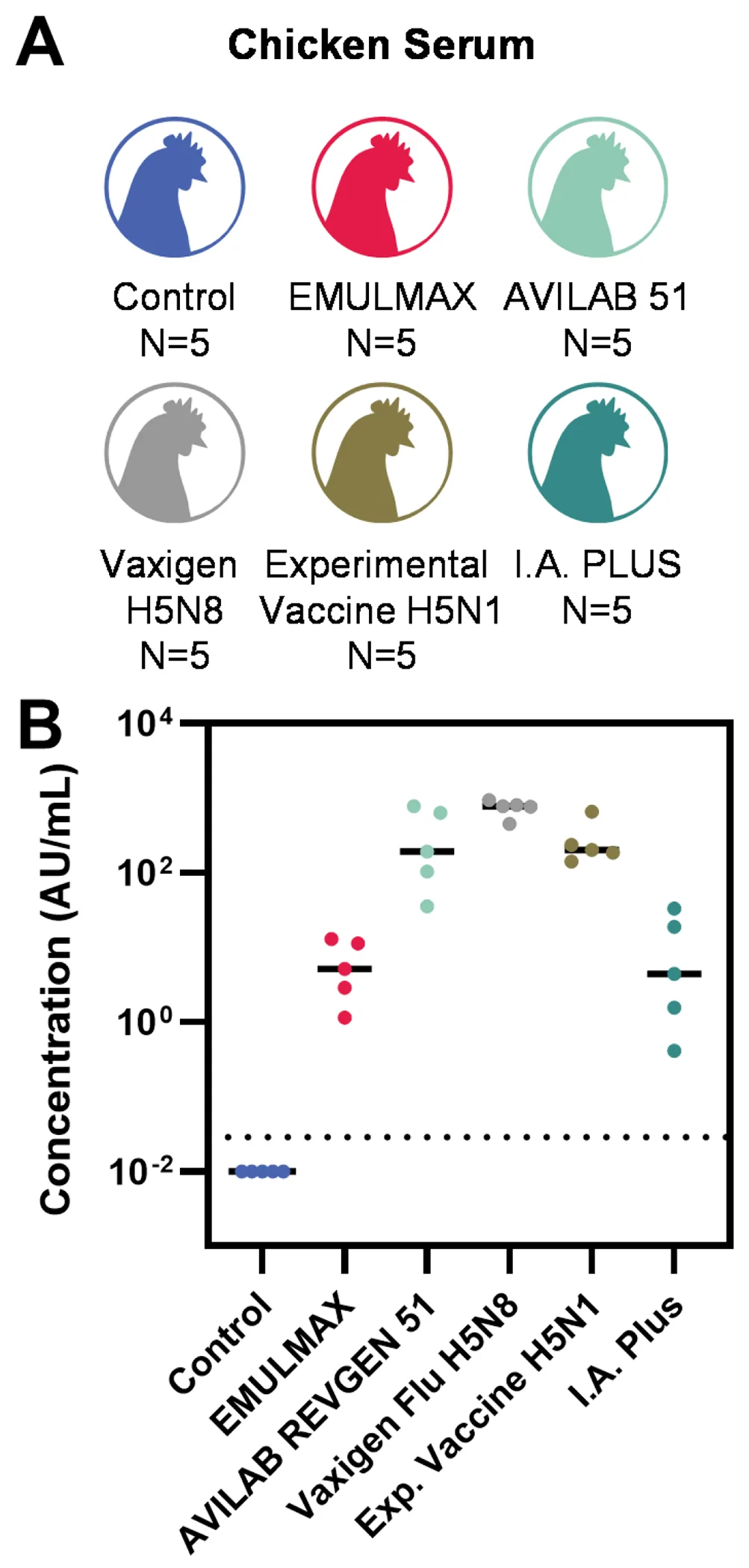
Vaccinated chicken serum contains H5-specific antibodies. (A) The sample cohort includes 5 healthy control serum samples and 25 H5N1-vaccinated chicken serum samples (5 vaccine types with 5 individuals per group). (B) Concentration of H5-specific antibodies. Dots represent individual values and solid horizontal lines represent the median for that group. The horizontal dashed lines are the cut-off values (2 x LOD) used to identify positive samples.

**Figure 5:**
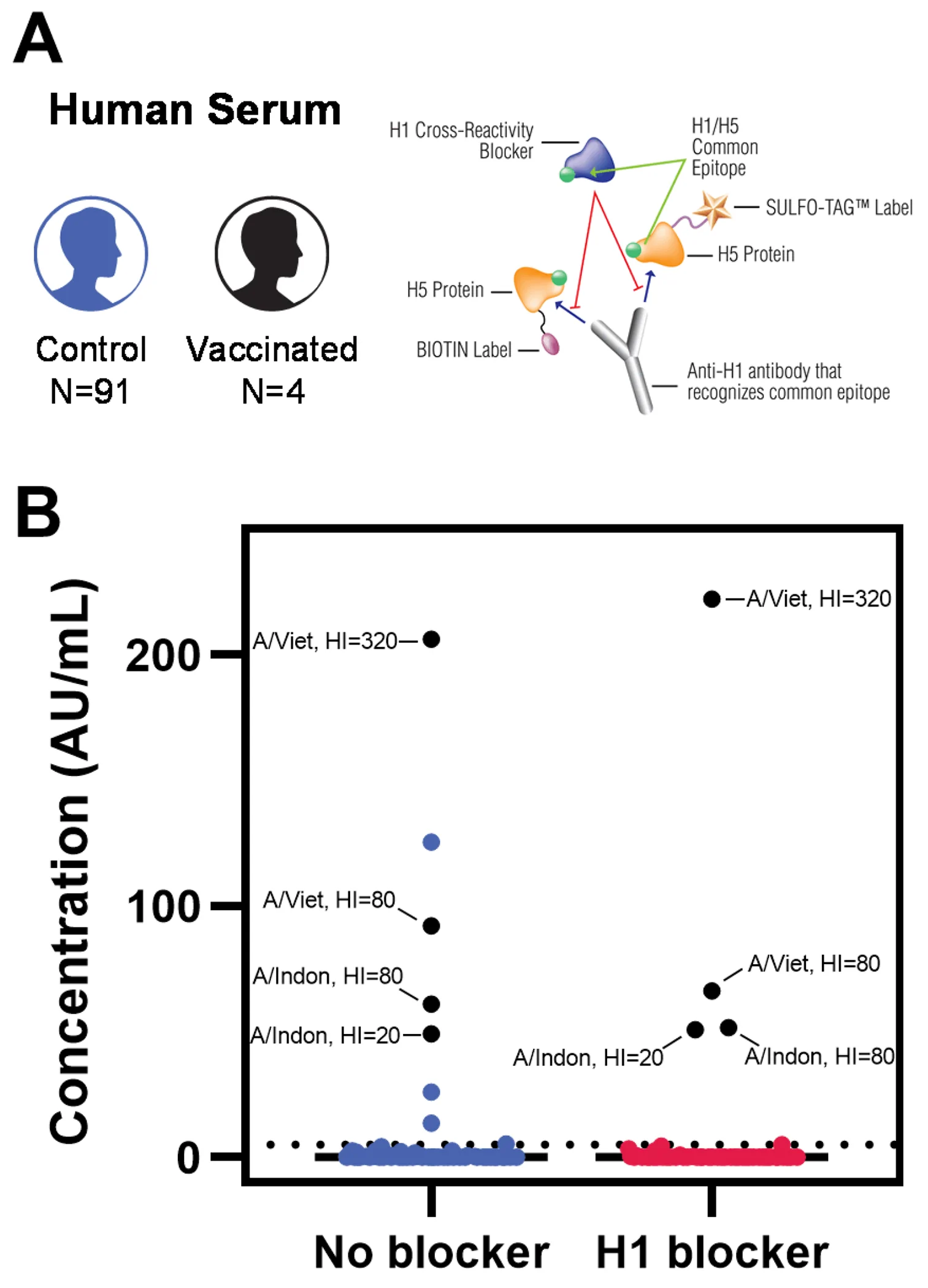
Vaccinated human serum contains H5-specific antibodies. (A) The sample cohort of in the study includes 91 healthy control serum samples and 4 H5N1-vaccinated human serum samples. The H1 Blocker is an H1 analog that is added to diluent for samples that have seroconverted to H1. (B) Concentration of H5-specific antibodies in samples from vaccinated (black) or presumed naïve samples. Samples were tested without H1 blocker (blue) and with H1 blocker (red). Vaccine strain and vendor-provided HAI titers are indicated in the text next to the sample icons. Dots represent individual values and solid horizontal lines represent the median for that group. The horizontal dashed line represents a potential assay threshold (5 AU/mL) that completely separates negative and positive samples for assays run in the presence of the H1 blocker.

## Data Availability

All data generated during this study are provided in this published article.
